# Lateral Scapular Winging and Shoulder Pain Following Posterior Fossa Surgery: A Case Report

**DOI:** 10.7759/cureus.112275

**Published:** 2026-07-08

**Authors:** Michael Collins, Dustin Harris

**Affiliations:** 1 Emergency Medicine, The University of Texas Southwestern Medical Center, Dallas, USA

**Keywords:** atypical presentation shoulder pain, physical exam, scapular winging, sports medicine, sports ultrasound

## Abstract

The spinal accessory nerve (SAN) provides motor innervation to the sternocleidomastoid and trapezius muscles. Injury to the distal segment of this nerve can result in trapezius denervation, leading to lateral scapular winging (LSW), shoulder pain, and impaired arm elevation. While iatrogenic SAN injury most commonly occurs during cervical lymph node biopsies and neck dissections, it may also arise following posterior fossa neurosurgical procedures due to prolonged traction, compression, or mechanical stress during surgical positioning or, less commonly, accidental direct ligation. Recognizing this complication is essential, as symptoms may mimic intrinsic shoulder pathology and delay appropriate diagnosis. A 36-year-old man presented with right shoulder pain, perceived instability, and scapular protrusion beginning shortly after posterior fossa brain tumor resection performed in the prone position. Physical examination revealed right-sided scapular winging with inferior and lateral scapular displacement and shoulder asymmetry. MRI of the right shoulder demonstrated trapezius edema and fatty streaking consistent with subacute denervation, along with mild rotator cuff tendinosis and a labral tear. Electromyography (EMG) and nerve conduction studies (NCS) confirmed severe right spinal accessory mononeuropathy with active denervation, though the nerve remained in continuity. Ultrasound (US) corroborated trapezius atrophy and loss of muscle architecture on the affected side. This case illustrates SAN injury as an underrecognized complication of posterior fossa surgery, likely resulting from positioning-related traction or compression. The clinical and electrodiagnostic findings localized the injury distal to the sternocleidomastoid branch, consistent with classical LSW. A distinction between lateral and medial scapular winging is reviewed: LSW results from trapezius dysfunction and is accentuated by arm abduction, whereas medial scapular winging results from serratus anterior weakness and is accentuated by forward flexion. Management begins with physical therapy targeting compensatory scapular stabilizers, with microsurgical repair or tendon transfer procedures reserved for refractory cases.

Clinicians should maintain a high index of suspicion for SAN injury in patients presenting with new shoulder pain or scapular winging following posterior fossa surgery, as early recognition facilitates timely rehabilitation and improved functional outcomes.

## Introduction

The spinal accessory nerve (SAN), also known as cranial nerve XI, provides motor innervation to the sternocleidomastoid and trapezius muscles [1]. Injury to this nerve can result in shoulder dysfunction characterized by pain, shoulder droop, weakness with arm elevation, and scapular winging due to trapezius denervation [2,3]. Because the SAN has a long superficial course in the posterior cervical triangle, it is particularly vulnerable to iatrogenic injury [4,5].

The most common etiologies of SAN injury include iatrogenic injury during surgical procedures, particularly cervical lymph node biopsies or neck dissections, which historically account for the majority of cases [6,7]. Other reported causes of SAN injury include blunt or penetrating trauma to the neck, compression injuries, traction injuries during surgical positioning, and tumor invasion or radiation-induced neuropathy [6,8]. Less commonly, SAN injury may occur following procedures involving the skull base or posterior fossa, such as the injury in the presented case [2,6,9,10].

Posterior fossa neurosurgical procedures may predispose patients to SAN injury through several mechanisms [2,9,10]. During these operations, patients are frequently placed in prone, park-bench, or lateral positions, which can place the shoulder girdle and cervical soft tissues under prolonged traction or compression [4]. The SAN is especially susceptible to stretch injury where it runs superficially between the sternocleidomastoid and trapezius muscles in the posterior cervical triangle [3]. Furthermore, surgical manipulation near the jugular foramen or upper cervical region may expose the nerve to mechanical stress or ischemia [2]. Prolonged operative time, compression by positioning devices, and shoulder depression to optimize surgical exposure may further increase the risk of neuropraxia or axonal injury [6]. Interestingly, many patients with SAN injury show an intact nerve with intraneural fibrosis when reexplored, which shows that the nerve does not need to be transected to result in significant dysfunction [4].

Injury to the SAN results in trapezius dysfunction, leading to impaired scapular stabilization, scapular winging, shoulder droop, and difficulty with shoulder abduction above shoulder level. Patients often present with shoulder pain and perceived instability, symptoms that may initially be mistaken for intrinsic shoulder pathology [2,3].

We present a case of SAN mononeuropathy resulting in scapular winging following posterior fossa brain tumor resection, as well as a discussion on the causes and characteristics of the different forms of scapular winging.

## Case presentation

The patient, a 36-year-old male with a history of brain tumor resection and current radiation therapy, presented to the clinic with persistent right shoulder pain. He reported that the pain began shortly after his brain surgery two months prior, with a progressive increase in discomfort upon movement of the shoulder. He described the shoulder as feeling lower than the opposite side with an associated sensation of instability in that same shoulder, expressing some concern that he felt like his shoulder “wanted to dislocate”. He also noted popping and cracking sounds with movement. 

He had not tried any medications for the pain and likewise denied any prior history of shoulder injuries, falls, or accidents. His discomfort was not present at rest, and he was able to continue work at his office job, spending long hours on the computer. Notably, the patient noticed that his scapula on the affected side protruded, a change he had not observed before his surgery less than two months prior. The surgical procedure lasted approximately 3.5 to four hours, during which he recalls being positioned prone on his chest. The exact positioning is unclear from the operation note, but classical neurosurgical prone positioning includes moderate neck flexion with the arms at the patient's side [11]. These symptoms, along with their emergence shortly after his surgery, prompted concern of nerve damage, particularly in relation to the shoulder's instability, possible scapular winging, and shoulder muscle weakness. 

**Figure 1:**
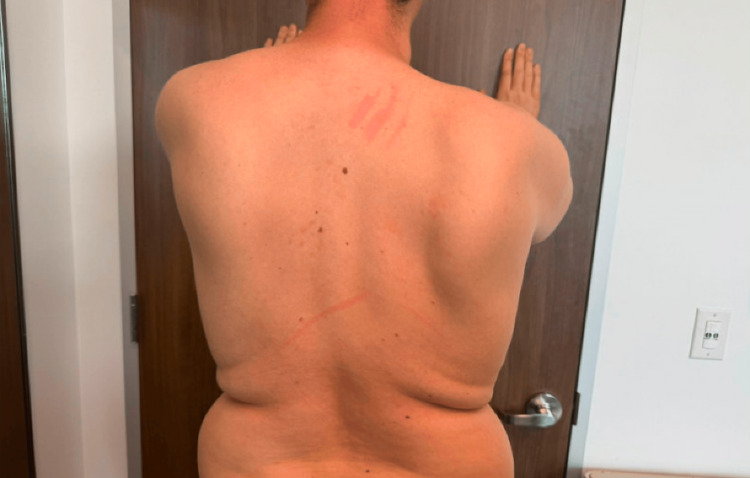
Photo of the patient's back five months post-op performing the wall-push/push-up test. Note the lateral and inferior displacement of the scapula and asymmetry of the shoulders. Pertinent exam findings: lateral and inferior displacement of the scapula; asymmetry of the shoulders, right inferior compared to the left; tender to palpation over the trapezius, rhomboids, anterolateral rotator cuff, and acromion; normal range of motion; O'Brien's test: positive; Neer's, Hawkins-Kennedy, Empty Can, Drop Arm, and Apprehension Test: negative

A physical exam confirmed scapular winging on the right, along with shoulder asymmetry, with the right shoulder being lower than the left (Figure 1). The shoulder was diffusely tender to palpation over the trapezius, rhomboids, anterolateral rotator cuff, and acromion. A recent MRI of his neck revealed mild disc bulges, but no other significant findings were reported. Additionally, his X-ray done the day of the first clinic visit, as well as previously done blood work, were all normal, and he has no other known medical history beyond the brain mass. 

Diagnostic workup

**Figure 2:**
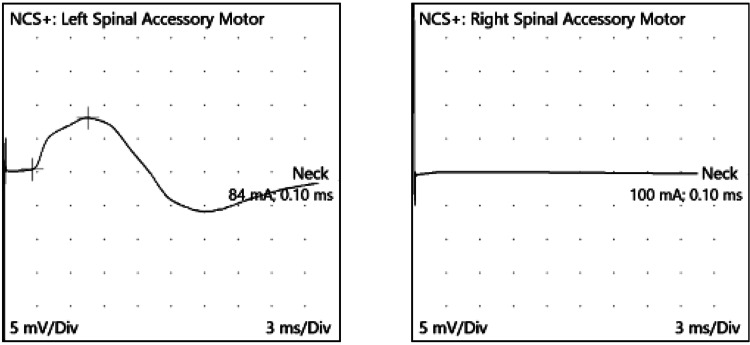
Nerve conduction studies (NCS) of the right and left spinal accessory nerve (SAN); left SAN response is normal, but the right SAN shows no appreciable response.

**Figure 3:**
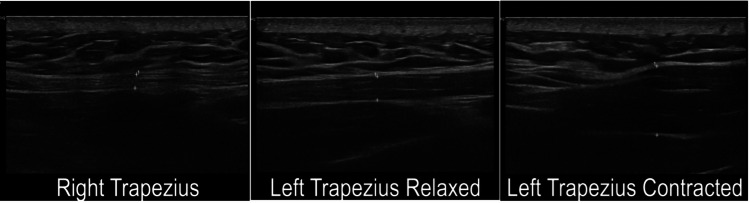
Ultrasound (US) images of the patient's trapezii; only one image of the smaller, atrophied right trapezius was included, as there was no change with volitional contraction. The left trapezius shows normal growth in size when contracted.

In the interval between the patient’s first and second visits, he received an MRI of the right shoulder along with electromyography (EMG)/nerve conduction studies (NCS) that also included an ultrasound (US) of the right upper extremity. Upon that second visit, roughly four months post-op, the patient reported that his shoulder discomfort had improved but that his shoulder function had not significantly changed. The MRI was significant for edema and fatty streaking of the trapezius, which was read as being concerning for subacute denervation. The MRI also showed signs of mild rotator cuff pathology with tendinosis of the supra- and infraspinatus along a mild labrum tear and acromioclavicular joint arthrosis. EMG/NCS confirmed the concern for denervation with “severe right spinal accessory mononeuropathy, with active denervation,” but did mention the nerve was in continuity (Figure 2). Additionally, the ultrasound performed in the same visit as the EMG/NCS showed right trapezius hyperechogenicity, atrophy, and loss of muscle architecture with no change in diameter with volitional contraction (Figure 3). The left side was normal on the US.

Management

There are a few management options that exist for SAN injury, but most regimens begin conservatively with physical therapy focused on strengthening the remaining muscles around the shoulder with action on the scapula, particularly the levator scapulae, rhomboids, and serratus anterior [2,3]. For patients who don’t improve, microsurgical repair of the SAN has been found to be effective up to a year from injury [7]. For patients for whom direct nerve repair is not an option or has failed, the Eden-Lange procedure is a proven surgical option. The procedure involves transferring the insertions of the levator scapulae, rhomboideus minor, and rhomboideus major muscles and can relieve pain, correct deformity, and improve function [2,3]. 

## Discussion

The patient’s clinical presentation, imaging findings, and electrodiagnostic results point toward a SAN injury. The patient exhibited no signs or symptoms of sternocleidomastoid denervation, and so it can be surmised that the injury occurred distally to the branch that innervates the sternocleidomastoid [1,12]. Such an injury often results in classical lateral scapular winging (LSW) [2,3,13].

Scapular winging is characterized by abnormal protrusion of the scapula due to weakness or paralysis of the muscles that stabilize it, particularly the serratus anterior and, similar to this case, the trapezius muscles [13]. Scapular winging can be categorized into medial and lateral forms, depending on the underlying nerve and muscle involved [13,14]. 

Classical LSW, as in the case above, is caused by SAN injury and thereby trapezius weakness or inherent trapezius muscle dysfunction. This compromises the trapezius’ role in scapular elevation and retraction, leading to the classic findings of the protrusion of the lateral border of the scapula along with rotation of the inferior angle medially and a scapula that sits lower and more protracted [3]. As discussed above, the etiologies of LSW are dominated by iatrogenic causes: posterior fossa surgery, as in this case; suboccipital decompression of Chiari malformations, and, most commonly, posterior cervical triangle surgeries [6,15]. Of note, LSW can also be caused by dorsal scapular nerve injury with associated rhomboid muscle weakness but results in a milder winging, with the scapula sitting higher and more laterally [16,17]. This is markedly less common than the other two forms of scapular winging, and, as such, any further mention of LSW will be referring only to classical SAN/trapezius-dysfunction-mediated LSW. 

In contrast, as further highlighted in Table 1, medial scapular winging (MSW) is classically caused by injury to the long thoracic nerve (LTN), which innervates the serratus anterior muscle, or injury to the muscle itself [18]. The serratus anterior helps to hold the scapula against the rib cage, and its weakness results in medial winging. A physical exam typically shows protrusion of the medial border of the scapula along with displacement of the inferior angle medially and posteriorly [5]. Neuralgic amyotrophy (NA) is the most common documented cause of MSW following trauma [15,19]. However, the difference between NA and trauma can be hard to delineate, as there is considerable overlap between those two categories. Repetitive stress from activities such as bench press and combat sports is considered NA; compression from a backpack would be considered trauma [16, 17, 19, 20]. Notably, iatrogenic causes are comparatively very rare [20]. 

**Table 1:** Comparison of the two most common forms of scapular winging

Feature	Medial Winging (Long Thoracic Nerve/Serratus Anterior)	Lateral Winging (Spinal Accessory Nerve/Trapezius)	References
Scapular position at rest	Medial border protrudes; inferior angle displaced medially and posteriorly	Lateral border protrudes; inferior angle rotates medially; scapula sits lower and more protracted	[13]
Winging accentuated by	Forward arm flexion (pushing against wall at 90°); winging was most prominent at 60-90° elevation	Arm abduction (especially >90°); overhead activities	[13]
Shoulder shrug	Normal (trapezius intact)	Weak or absent	[13, 16]
Arm elevation pattern	Difficulty with forward flexion; cannot fully elevate arm anteriorly	Difficulty with abduction; limited overhead reach	[16]

Patients with MSW struggle with forward flexion and classically cannot fully elevate their arm anteriorly but have a normal shoulder shrug, as their trapezius is intact [18]. Conversely, LSW patients have trouble with lateral shoulder abduction and overhead reach and have a weak or absent shoulder shrug classically, although C3 and C4 nerve root innervation commonly (up to 95%) preserves some shoulder shrug ability [12]. MSW is accentuated by forward arm flexion, especially when pushing against a wall at a 60-to-90-degree angle, whereas LSW is accentuated by arm abduction, especially in the lateral plane above 90 degrees and with overhead activities [21]. 

## Conclusions

This case highlights the importance of recognizing SAN injury as a complication of posterior fossa surgery, especially in patients with new shoulder pain or weakness in the immediate postoperative period. The LSW observed in this patient is directly related to SAN injury and subsequent denervation of the trapezius muscle. Understanding the differences between MSW and LSW is essential in guiding appropriate diagnostic and therapeutic interventions. Given the patient's ongoing recovery and evidence of axonal regrowth, continued monitoring and rehabilitation are key to optimizing his functional recovery. 
